# Current Systemic Treatment Options for Advanced Pancreatic Cancer—An Overview Article

**DOI:** 10.3390/biomedicines14010188

**Published:** 2026-01-15

**Authors:** Małgorzata Domagała-Haduch, Anna Długaszek, Anita Gorzelak-Magiera, Iwona Gisterek-Grocholska

**Affiliations:** Department of Oncology and Radiotherapy, Medical University of Silesia, 40-514 Katowice, Poland; aniakrywult@gmail.com (A.D.); anitagor@op.pl (A.G.-M.); igisterek@sum.edu.pl (I.G.-G.)

**Keywords:** advanced pancreatic cancer, chemotherapy, targeted therapy

## Abstract

Pancreatic adenocarcinoma is one of the most aggressive malignancies, with a steadily increasing incidence rate. Due to the asymptomatic nature of early cancer and frequent late diagnosis, only 10–20% of patients are considered for radical treatment. In approximately 40% of patients, local advancement precludes primary surgical treatment, and in approximately half of patients, the cancer is diagnosed at the metastatic stage. Treatment of advanced pancreatic cancer is based on systemic therapy, while a growing number of studies are focusing on the potential use of molecularly targeted agents. The median survival time for metastatic patients treated with FOLFIRINOX chemotherapy is 11 months, compared to 8.5 months for patients treated with gemcitabine and nab-paclitaxel-based chemotherapy. Olaparib in the maintenance treatment of patients with advanced pancreatic cancer prolongs the time to progression compared to placebo but does not affect median overall survival. Immunotherapy and targeted therapy have so far been used in a narrow group of patients with a specific molecular profile, but further research on this cancer offers a real opportunity to develop new treatment approaches. This review article is based on the NCCN (National Comprehensive Cancer Network) guidelines and publications available in the PubMed database.

## 1. Introduction

Each year, 460,000 people worldwide are diagnosed with pancreatic cancer, and over 430,000 patients die from the disease annually, including 120,000 in Europe [1,2].

According to American epidemiological data, pancreatic cancer accounts for approximately 3% of all malignant tumors and 8% of all cancer deaths. It is estimated that by 2030, pancreatic cancer will become the second leading cause of cancer deaths. It is currently the third leading cause of death among women (after lung and breast cancer) and the fourth leading cause of death among men (after lung, prostate, and colon cancer) [3,4].

The most important risk factors for developing pancreatic cancer include cigarette smoking and exposure to secondhand smoke. In individuals with long-term exposure to secondhand smoke during childhood, the risk of developing pancreatic cancer is 2.6 times higher, while in individuals exposed to secondhand smoke only in adulthood, the risk is 1.54 times higher [5].

The association between obesity and an increased incidence of pancreatic cancer has been documented in several meta-analyses. These studies indicate that excess body weight is a relative risk factor for developing this cancer, estimated at approximately 1.5 times greater [6,7]. Among metabolic causes, besides obesity, the role of type 2 diabetes is also emphasized as another risk factor for pancreatic cancer [8].

Family history is another risk factor for pancreatic cancer, occurring in 5–10 percent of patients [1]. Prospective studies have shown that the occurrence of pancreatic cancer in a first-degree relative in the case of a family history of pancreatic cancer is associated with a nine-fold risk of developing the disease [9].

Only 10% of pancreatic cancer cases are caused by genetic disorders [1], which primarily include Peutz–Jeghers syndrome (mutations in the STK11 tumor suppressor gene), mutations in the BRCA genes, the PALB gene, and the MLH1 and MSH2 genes (defects occurring in Lynch syndrome). Other genetic mutations (defects in the PRRS1 or p16/CDKN2A genes) are reported sporadically.

Currently, the standard treatment for patients with pancreatic cancer is testing for germline mutations in the BRCA1/BRCA2 genes. The presence of this mutation is associated with increased sensitivity to platinum-based chemotherapy and is a predictor of response to olaparib therapy. Furthermore, the detection of this mutation necessitates initiating genetic testing of the patient’s relatives [10,11].

Pancreatic cancer is a mildly symptomatic disease, and in its early stages, patients are asymptomatic. In more advanced stages, abdominal and/or back pain (70–80%), weight loss (90%), fatigue (50%) and diarrhea (27%) most often occur [12].

In some patients, decompensation of diabetes or the development of new diabetes may be a telltale sign of pancreatic cancer. Occasionally, a paraneoplastic syndrome such as thrombophlebitis (Trousseau syndrome) or acanthosis nigricans is a harbinger of pancreatic cancer [13,14]. The presence of ascites always indicates advanced disease.

Advancement of the disease is the most important prognostic factor, assessed according to the UICC (Union for International Cancer Control) classification with TNM staging [15]. From a clinical perspective, the most important classification is the possibility of radical surgery, as it is the only method offering a chance of long-term survival. Due to the asymptomatic nature of early cancer, only 10–20% of patients can undergo surgical treatment, and 40% of patients are diagnosed at stage IV. In 40–50% of patients, the cancer is locally advanced, preventing primary surgical treatment with curative intent [16].

Among the prognostic factors, Ca19.9 antigen remains one of the most important—its concentration is an indirect indicator of tumor resectability: values below 100 U/mL indicate a high probability of resectable disease, while higher values are associated with unresectable and metastatic tumors. A decrease in Ca19.9 antigen concentration during palliative therapy translates into prolonged overall survival. Biomarker assessment has certain limitations resulting from the sensitivity of the method (80%) and the lack of secretion of this protein by a few percent (5–10%) of the population; excessive Ca19.9 concentrations occur in obstructive jaundice, and then the marker concentration does not correspond to the stage of the cancer [17].

## 2. First-Line Treatment for Advanced Pancreatic Cancer

For patients in good or very good general condition, the treatment of choice is the FOLFIRINOX regimen (5-fluorouracil in a 46 h continuous infusion of 2400 mg/m^2^, 5-fluorouracil bolus of 400 mg/m^2^, oxaliplatin 85 mg/m^2^, irinotecan 180 mg/m^2^) administered every 14 days. This regimen utilizes the additive action of an antimetabolite, a topoisomerase I inhibitor, and an alkylating agent.

In a phase III clinical trial enrolling 342 patients with advanced pancreatic cancer, FOLFIRINOX chemotherapy significantly prolonged median OS and PFS compared to gemcitabine-based chemotherapy (1000 mg/m^2^ every 7 days for 7 consecutive weeks, then on days 1, 8, and 15 every 28 days). In the study group, median OS was 11.1 months and PFS was 6.4 months, while in the control group, median OS was 6.8 months and PFS was 3.3 months (*p* < 0.001). It should be noted that FOLFIRINOX chemotherapy demonstrated higher treatment toxicity, primarily in the hematologic adverse events group: neutropenia was reported in 46% of patients, febrile neutropenia occurred in 5% of patients, and 42.5% of patients required granulocyte growth factor therapy. In the control group, these percentages were 21%, 1%, and 5%, respectively. Among non-hematological adverse events, weakness, diarrhea, vomiting, and polyneuropathy were more frequently reported in the study group. Despite the higher toxicity, FOLFIRINOX chemotherapy significantly delayed deterioration in quality of life—after 6 months of therapy, this occurred in 31% of patients in the study group and in 66% of patients in the control group [18].

In patients with contraindications to the triple-drug regimen, gemcitabine with nab-paclitaxel is recommended.

Paclitaxel is a drug that stabilizes microtubules by inhibiting depolymerization and, consequently, mitosis, ultimately leading to cell death. Nab-paclitaxel contains paclitaxel bound to human albumin nanoparticles. This combination facilitates drug transport across endothelial cells.

The efficacy of the combination of nab-paclitaxel and gemcitabine in patients with metastatic pancreatic cancer was demonstrated in the MPACT study, which enrolled 861 patients.

Patients in the study group received dual therapy: nab-paclitaxel 125 mg/m^2^ and gemcitabine 1000 mg/m^2^ on days 1, 8, and 15 (every 28 days); the control group received gemcitabine monotherapy at a dose of 1000 mg/m^2^ every 7 days for 7 consecutive weeks, then on days 1, 8, and 15 (every 28 days).

The median OS was 8.5 months in the study group and 6.7 months in the gemcitabine monotherapy group (*p* < 0.001). A longer median PFS was also achieved—5.5 vs. 3.7 months (*p* < 0.001). It should be noted that dual therapy was associated with higher toxicity—neutropenia, weakness, and peripheral neuropathy were more common in the study group [19].

Currently, there is no data available from large randomized clinical trials comparing the efficacy of the two regimens described above.

A retrospective comparative analysis of over 1000 patients with advanced pancreatic cancer, published in 2022, demonstrated a difference in median OS in favor of triplet chemotherapy (9.3 vs. 6.9 months, *p* < 0.001). Furthermore, in this study, the FOLFIRINOX regimen was associated with a lower rate of treatment-related complications. The presented data, particularly regarding the reduced toxicity of triplet therapy, should be interpreted with caution, as patients treated with the nab-paclitaxel-containing regimen had a higher median age, poorer performance status, and greater comorbidity, which likely influenced the analysis results [20].

Hyperbilirubinemia is a common clinical problem in patients with advanced pancreatic cancer. Even with successful biliary stenting, elevated bilirubin levels persist for up to several weeks. In such aggressive cancer, waiting for bilirubin to normalize may be associated with the risk of progression and deterioration in performance status. Therefore, it is reasonable to consider safe treatment options. In the presence of hyperbilirubinemia, palliative chemotherapy based on the paclitaxel-gemcitabine regimen appears to be safe. In one study, the use of chemotherapy based on the above-mentioned drugs in patients with hyperbilirubinemia did not increase treatment toxicity or adverse events [21].

The use of a regimen containing irinotecan is not recommended due to its hepatic toxicity. However, study results indicate that chemotherapy based on oxaliplatin and 5-fluorouracil is a safe treatment option [22].

In patients with poorer performance status (WHO 2-3) and/or significant comorbidities, gemcitabine monotherapy (at a dose of 1000 mg/m^2^ every 7 days for 7 consecutive weeks, then on days 1, 8, and 15 every 28 days) should be considered. In a study comparing the efficacy of gemcitabine with 5-fluorouracil (at a dose of 600 mg/m^2^ once weekly), median overall survival increased from 4.4 to 5.6 months, and the objective response rate increased from 0% to 5.4% (*p* = 0.0025). Gemcitabine treatment is generally well tolerated and does not impair quality of life [23].

Irinotecan is a camptothecin derivative, an inhibitor of topoisomerase I (an enzyme involved in DNA replication). Irinotecan and its metabolite SN-38 irreversibly block the DNA-topoisomerase complex, inducing cytotoxic effects. The liposomal form of irinotecan has been shown to have higher plasma concentrations and a prolonged presence of the SN-38 metabolite in the tumor region compared to the traditional molecule.

In the Napoli-3 study, 383 patients received the NALIRIFOX regimen (liposomal irinotecan 50 mg/m^2^, oxaliplatin 60 mg/m^2^, leucovorin 400 mg/m^2^, and 5-fluorouracil 2400 mg/m^2^ administered over 46 h on days 1 and 15 of a 28-day cycle). The control group (387 patients) received gemcitabine and nab-paclitaxel (gemcitabine 1000 mg/m^2^ and nab-paclitaxel 125 mg/m^2^ on days 1, 8, and 15 of a 28-day cycle).

NALFIRINOX chemotherapy resulted in a median OS of 11.1 months, compared to 9.2 months in the group receiving doublet chemotherapy (*p* = 0.036) and prolonged median PFS (7.4 vs. 5.6 months, *p* < 0.0001). Chemotherapy in both groups demonstrated significant toxicity, with adverse events of at least grade 3 (mainly diarrhea, hypokalemia, nausea, and neutropenia) reported in nearly 90% of patients [24].

NALIRIFOX is recommended for patients with advanced pancreatic cancer in good general condition but is currently not a widely used regimen due to cost and the lack of a clear benefit in overall survival compared to FOLFIRINOX chemotherapy.

In patients with confirmed germline BRCA1 or BRCA2 mutations and no progression of disease after at least 16 weeks of platinum-based chemotherapy, the use of olaparib (a polyADP ribose polymerase inhibitor) as maintenance therapy allows for a longer median PFS compared to placebo (median PFS 7.4 vs. 3.8 months) [25].

Patients in poor general condition do not benefit from systemic therapy. In this group, symptomatic treatment with palliative radiotherapy is recommended.

## 3. Second-Line Treatment

Patients with satisfactory performance status who have experienced cancer progression during first-line treatment are candidates for second-line systemic therapy.

In patients with progression treated with FOLFIRINOX, gemcitabine remains the standard of care. This approach, according to data from small-group studies, results in a median OS of 5.7 months, but there are no studies in large groups of patients regarding the efficacy of this treatment [26].

Reports on the efficacy of the combination of gemcitabine and nab-paclitaxel are scarce, but preliminary data demonstrate the benefit of using this doublet.

A phase II study evaluating the efficacy of second-line treatment with gemcitabine and nab-paclitaxel in 40 patients with advanced pancreatic cancer who progressed during FOLFIRINOX chemotherapy achieved a median PFS of 5.8 months and a median OS of 9.9 months.

Grade ≥ G3 treatment-related toxicity occurred in over 60% of patients and was primarily associated with hematological toxicity [27].

In a retrospective multicenter study, Zaibet et al. collected treatment outcomes for 427 patients. After cancer progression during FOLFIRINOX treatment, patients receiving gemcitabine plus nab-paclitaxel achieved longer median OS (7.5 vs. 4.7 months) and PFS (3.5 vs. 2.3 months), as well as a higher disease control rate (DCR)—56% vs. 32%, compared to gemcitabine monotherapy [28].

In 2025, treatment results from multiple oncology centers in Turkey were compiled: data from 218 patients treated with gemcitabine and nab-paclitaxel after progression during FOLFIRINOX therapy were analyzed. It was shown that in the gemcitabine and nab-paclitaxel group, the median PFS was 5.1 months and the median OS was 8.6 months [29].

The use of gemcitabine with conventional paclitaxel (GEMPAX regimen) did not translate into improved survival in patients after failure of first-line treatment. A phase III study conducted in 211 patients demonstrated that dual-drug therapy, compared to gemcitabine monotherapy, increased the response rate (ORR) in the study group (17.1% vs. 4.2%, *p* = 0.008) and translated into a slight prolongation of PFS (3.1 vs. 2 months, *p* = 0.0067). Median OS in the study group was 6.4 months, and in the control group, 5.9 months—however, this parameter did not reach statistical significance (*p* = 0.4); hence, the study is considered negative [30].

In the case of progression during first-line chemotherapy with gemcitabine and nab-paclitaxel, patients in good general condition may be considered for triple or doublet chemotherapy with 5-fluorouracil and oxaliplatin. However, data from large clinical trials on the efficacy of such treatment are lacking. The literature contains reports from studies with smaller groups of patients, without the use of a comparator.

In a study conducted on 104 patients who progressed during chemotherapy with gemcitabine and nab-paclitaxel, they received a modified Folfirinox regimen (omitting the 5-fluorouracil bolus and reducing the irinotecan dose to 150 mg/m^2^); the median OS was 7 months, and the median PFS was 3.9 months. Grade 3 or higher toxicity occurred in more than half of the treated patients [31].

In a similar study conducted in a Japanese population, the use of the FOLFIRINOX regimen resulted in slightly better treatment outcomes—median OS was 12.1 months and median PFS was 5.3 months. The researchers found no difference in the efficacy of the triplet regimen compared to its modified version. This analysis is limited by the small sample size—23 patients were enrolled in the study [32].

The randomized phase III CONCO-003 study analyzed data from 160 patients who had failed gemcitabine therapy and were treated with second-line chemotherapy based on 5-fluorouracil or in combination with oxaliplatin. The addition of oxaliplatin resulted in an extension of the median OS from 3.3 months to 5.9 months in the doublet regimen group. The rate of adverse events was comparable in both subgroups, with the exception of polyneuropathy, which was more common in the two-drug regimen group [33].

Opposite results were obtained in the phase III PANCREOX study, which included data from 108 patients treated with the FU/LV (5-fluorouracil with leucovorin) or FOLFOX6 regimen. Patients treated with the oxaliplatin-containing regimen experienced a shorter median OS (6.1 vs. 9.9 months, *p* = 0.02) and a significantly higher rate (63%) of G3-G4 complications. The increased adverse events, mainly neutropenia and fatigue, were most likely due to the high doses of fluorouracil (400 mg/m^2^ bolus followed by a 46 h infusion of 5-fluorouracil at a dose of 2400 mg/m^2^)—significantly higher than in the previously cited study [34].

Oxaliplatin therapy with 5-fluorouracil (the most commonly used regimen is FOLFOX4 with 5-fluorouracil doses of 400 mg/m^2^ in a bolus followed by a 46 h infusion of 1200 mg/m^2^ of 5-fluorouracil—adapted from the CONCO3 study and modified for clinical practice) appears to be a rational choice for patients in good or relatively good performance status (Karnofsky score ≥ 70) after failure of gemcitabine.

Liposomal irinotecan has been shown to be effective in patients with progression after gemcitabine-based chemotherapy. The phase III NAPOLI-1 study included 417 patients; patients in the study group received liposomal irinotecan at a dose of 120 mg/m^2^ every 21 days, and those in the control group received 5-fluorouracil at a dose of 200 mg/m^2^ in combination with leucovorin at a dose of 200 mg/m^2^ every week, followed by a two-week break after four courses. During the study, the study was modified, and a third group was added, receiving liposomal irinotecan at a dose of 80 mg/m^2^ in combination with 5-fluorouracil at 2400 mg/m^2^ and leucovorin at 400 mg/m^2^. The combination of liposomal irinotecan with 5-fluorouracil resulted in a prolonged median OS compared to 5-fluorouracil therapy (6.2 vs. 4.2 months, *p* = 0.002), whereas liposomal irinotecan monotherapy did not significantly improve OS compared to 5-fluorouracil therapy—median OS was 4.9 and 4.2 months, respectively. In the group treated with liposomal irinotecan with 5-fluorouracil, almost three-quarters of patients required dose modifications of cytotoxic drugs or even treatment discontinuation due to adverse events; in the group treated with liposomal irinotecan monotherapy, this percentage was 56%, and in the control group, 37%. The most common adverse events in the study group were neutropenia and diarrhea. 12.8% of patients discontinued treatment due to toxicity [35].

## 4. Molecularly Targeted Therapy and Immunotherapy

A small number of pancreatic cancer patients experience microsatellite instability, and in these cases, treatment with programmed cell death receptor 1 inhibitors (anti-PD1) offers a chance for treatment response. In the phase II basket trial KEYNOTE 158u, 233 patients with solid tumors exhibiting high levels of microsatellite instability (MSI-H/dMMR) were treated with pembrolizumab at a standard dose of 200 mg/m^2^ after exhaustion of standard treatment options. Treatment was planned for 35 cycles or until disease progression. Objective response rate (ORR) was achieved by 34% of patients in the overall cohort, with a median OS of 23.5 months and a median PFS of 4.1 months. In the subgroup of patients with advanced pancreatic cancer (23 patients), one patient achieved a complete response and four achieved a partial response. The median PFS was 2.1 months, and the median OS was 4 months [36].

RAS (rat sarcoma virus) genes, which include KRAS (Kristen rat sarcoma viral oncogene homolog) and NRAS (neuroblastoma RAS viral oncogene homologue), are a well-known family of oncogenes [37]. Mutations in the RAS genes underlie the pathogenesis of many cancers, such as pancreatic, colon, and lung cancer [38]. Mutations in the KRAS gene are estimated to occur in 85–90% of pancreatic cancers [39]. However, until recently, developing drugs targeting this target seemed impossible.

After confirmation of the G12C mutation in the KRAS gene, sotorasib therapy improved treatment outcomes. In a Phase I/II study conducted in 38 patients who had failed at least one prior line of treatment, sotorasib resulted in a median PFS of 4 months and an OS of 6.9 months. An objective response to treatment was achieved in 21% of patients [40].

Unfortunately, KRAS G12C mutations constitute a small fraction of detected abnormalities, leaving a significant portion of pancreatic cancer patients without the option of targeted therapy.

Daraxonrasib (RMC-6236) is an oral, multiselective RAS protein inhibitor that works by blocking the interaction of the active RAS protein (RAS(ON)) with its target proteins, forming a so-called RAS-inhibitor-accessory protein “tri-complex,” leading to inhibition of intracellular signaling pathways [41]. Currently, the phase III RASolute 302 study (NCT06625320) is ongoing in patients with previously treated metastatic pancreatic adenocarcinoma with KRAS G12X mutations, comparing the efficacy of this therapy with standard chemotherapy [42]. Preliminary results from the phase I/II study showed a median PFS of 8.5 months in the KRAS G12X subgroup (n = 22) and 8.1 months in the broader RAS-mut group (n = 37). ORR was 35% in the G12X subgroup and 29% in the RAS-mut group. Disease control rates (DCR) were 92% and 95%, respectively. Median OS was 13.1 and 15.5 months, respectively, which translates to a significant improvement compared to standard gemcitabine/nab-paclitaxel chemotherapy (OS 7.4 months) [43]. In 2025, daraxonrasib received a positive FDA recommendation and breakthrough therapy status, but full verification of the drug’s benefits in a phase III trial and identification of optimal therapeutic combinations and resistance mechanisms remain necessary [44].

Another promising new drug is zenocutuzumab, a bispecific IgG1 antibody. By blocking the formation of HER2/HER3 dimers, this drug disrupts the PI3K/AKT/mTOR signaling pathway, which is crucial for cancer cell survival and proliferation. This drug has demonstrated efficacy in patients with the NGR1 gene fusion, present in approximately 1% of patients.

In a phase II trial involving 36 patients diagnosed with advanced pancreatic cancer, 42% achieved a treatment response.

In the entire group of patients with advanced solid tumors and NGR1 fusion, 30% of patients achieved a response to zenocutuzumab therapy, the median duration of response was 11.1 months, and the median survival time to disease progression was 6.8 months [45]. Despite promising results of the therapy, it will be possible to use it in a small number of patients with neuregulin fusion.

Currently, additional mutations potentially at risk of becoming emergency targets are being studied. Glycogen synthase kinase 3β is attracting attention, as it is considered a factor promoting tumor growth, metastasis, and the development of chemoresistance [46].

New therapies that influence the immune system, such as cancer vaccines and CAR-NKT cell therapy, also bring hope—however, there is currently no evidence from clinical trials indicating the effectiveness of such treatment.

## 5. Summary

Advanced pancreatic cancer has a very poor prognosis. Progress in the treatment of this cancer has been minimal compared to other cancers. The use of the FOLFIRINOX regimen as first-line treatment has extended the median overall survival by 5 months compared to the standard treatment regimen of the early 1990s, which was gemcitabine or 5-fluorouracil. In patients with germline mutations in the BRCA1 or BRCA2 genes treated with at least disease stabilization after 4 months of platinum-based therapy, maintenance therapy with olaparib may be considered, which allows for an extension of the median PFS compared to placebo. This drug does not prolong overall survival; however, its use is important in patients who do not tolerate systemic therapy well—it allows for maintaining the therapeutic effect achieved after chemotherapy without significantly affecting quality of life.

In patients with contraindications to triple therapy, the optimal regimen is the combination of nab-paclitaxel with gemcitabine. This treatment is characterized by a two-month improvement in overall survival compared to gemcitabine monotherapy, with acceptable side effects.

The choice of second-line regimen depends primarily on the patient’s clinical condition and the treatment used in first-line therapy. In clinical practice, gemcitabine monotherapy is most often used after progression during FOLFIRINOX therapy, although this may soon change due to the growing body of data confirming the greater efficacy of the combination of gemcitabine with nab-paclitaxel in second-line therapy.

After gemcitabine therapy failure, FOLFOX is the most commonly used regimen.

Studies of immunotherapy and molecularly targeted agents have not yielded breakthroughs in the treatment of this type of cancer—their effectiveness has been demonstrated in only a small percentage of patients. High hopes are pinned on daraxonrasib, a drug targeting KRAS G12x mutations, which are present in up to 90% of pancreatic cancer patients. The use of this drug could represent a breakthrough in treatment, but there are currently no study results from a representative sample of patients.

It should be emphasized that in patients with advanced pancreatic cancer, appropriate symptomatic treatment, including pain relief in accordance with WHO guidelines, is just as important as systemic treatment, including pharmacological and invasive therapies such as celiac plexus neurolysis or radiotherapy, as well as nutritional therapy and psychological support.

## Data Availability

No new data were created or analyzed in this study.
